# Foot Placement Modulation Diminishes for Perturbations Near Foot Contact

**DOI:** 10.3389/fbioe.2018.00048

**Published:** 2018-05-08

**Authors:** Mark Vlutters, Edwin H. F. Van Asseldonk, Herman van der Kooij

**Affiliations:** Department of Biomechanical Engineering, University of Twente, Enschede, Netherlands

**Keywords:** perturbed human walking, balance control, foot placement, extrapolated center of mass, capture point

## Abstract

Whenever a perturbation occurs during walking we have to maintain our balance using the recovery strategies that are available to us. Foot placement adjustment is often considered an important recovery strategy. However, because this strategy takes time it is likely a poor option if the foot is close to contact at the instant a perturbation occurs. The main goal of this study is to gain a better understanding of how humans deal with balance perturbations during walking if foot placement adjustments are constrained by time. Ten healthy subjects walked on an instrumented treadmill and received mediolateral and anteroposterior pelvis perturbations at various instances during the single support phase. The results show that foot placement modulation in the first recovery step following anteroposterior perturbations is fairly invariant of the perturbation magnitude and direction, regardless of the onset instance. For mediolateral perturbations, foot placement adjustments strongly modulate with the perturbation magnitude and direction, but these effects diminish when the perturbation onset is closer to the instant of foot contact. For most perturbations the first recovery step was consistent across subjects for all onset instances. However, in the second step various strategies arose that were not consistent across subjects, nor within subjects, especially for perturbations applied close to foot contact. Despite these different strategies, the COP location following foot contact strongly related to the COM velocity throughout these strategies. The results show that humans have various ways to compensate for limited availability of a foot placement strategy, with strategy selection highly dependent on the instant during the gait phase at which the perturbation is applied.

## Introduction

Human balance control is highly flexible, with a multitude of strategies that can be addressed to reject disturbances and allow continuation of walking. One example is the modulation of ankle joint moments to affect the movement of the body. Another are inertia-based strategies such as the hip strategy, in which changes in angular momentum are used to affect linear body motion. Furthermore, foot placement modulation can change the base of support area, allowing adjustments to be made to the walking cycle. This might be achieved by adjusting both the location and timing of foot placement. To gain more insight in human balance control and the preferred ways of balance recovery, it is helpful to understand how humans maintain balance when one or multiple strategies are restricted.

The way balance is controlled depends on physical capabilities and constraints. For example, experiments in standing balance show that humans no longer utilize an ankle strategy if the size of the support surface is decreased (Horak and Nashner, 1986). This makes the ankle strategy ineffective, and possibly even threatening to balance. In walking, we have previously shown that foot placement adjustments are elicited in response to anteroposterior (AP) perturbations after physically blocking the ankle joints to make an ankle strategy ineffective (Vlutters et al., 2018). Such adjustments in foot placement were not observed following AP perturbations in normal walking (Vlutters et al., 2016). Other changes to the physical capabilities of the body also modify balance control, such as increased body sway in unilateral amputees (Geurts et al., 1992), or enhanced lateral balance performance through the use of a powered ankle device (Kim and Collins, 2015).

Constraints on balance control can also be in the form of time. Especially for foot placement modulation, time is required to make adjustments to the swing leg (Hof et al., 2010). The instance of the gait cycle at which a disturbance occurs is therefore an important factor in determining how balance will be maintained. If a disturbance occurs shortly before foot contact, there is little time to make foot placement adjustments. As a result, adjustments might have to be postponed to the subsequent step, or other balance strategies have to be addressed to compensate. Especially mediolateral (ML) disturbances given briefly before foot contact are expected to be challenging, given the already limited availability of other strategies, such as ML ankle control.

In an attempt to make predictions of balance control strategies during gait, the center of mass (COM) velocity has previously been shown to relate to the center of pressure (COP) location following foot contact, in the first recovery step following both ML (Hof et al., 2007, 2010; Vlutters et al., 2016) and AP (Vlutters et al., 2016) perturbations. For AP perturbations, this COP shift was realized during the double support phase without the need to strongly adjust the location of the leading foot as compared to the unperturbed condition. For ML perturbations however, this COP shift was made possible mainly through foot placement adjustments. In addition, this COP shift was in line with the velocity-dependent extrapolated center of mass (XCOM) concept (Hof et al., 2005). This concept is also known as the capture point (Pratt et al., 2006), which can be derived from a linear inverted pendulum model. The XCOM can be represented as a point on the floor at a horizontal distance from the COM, equal to the COM velocity times a proportionality constant $\omega_{0}^{-\text{1}}$. If the model's COM moves toward the COP while the COP coincides with the XCOM, the model will come to an upright movement stop. If human walking has similarities to the motion of an inverted pendulum, the ability to balance and to steer the COM might be reflected by the ability to locate the COP relative to the XCOM. If there would be insufficient time to adjust the base of support through foot placement adjustments, it might not be possible to displace the COP in accordance with the XCOM, because the COP is constrained to the base of support. The COM could move in an undesired direction as a result, and the COM velocity would lose its predictive value for the COP in that step. It is unclear if such predictions would hold for the subsequent second step, especially if subjects cannot counteract the disturbances during the double support phase after the first step.

The main goal of this study is to gain a better understanding of how humans deal with balance perturbations during walking if foot placement adjustments are constrained by time. Specifically, for perturbations with an onset increasingly close to the instant of foot contact we question (1) whether foot placement adjustments diminish when there is little time to use such adjustments as a recovery strategy, and (2) whether the COP will continue to modulate with the COM velocity, in line with the XCOM? Foot placement adjustments are expected to diminish in the first recovery step given the time required to move the foot. However, strategies other than foot placement adjustments might still facilitate COP modulation with the COM velocity. Furthermore, there will be more time to make foot placement adjustments in the second recovery step, which might allow for such modulation. We investigate these questions by applying both ML and AP perturbations to the pelvis of human subjects walking on a treadmill, at various instances within the single support phase, and capturing their kinematics. We analyze the foot placement locations and COP positions relative to the COM at specific instances following perturbation onset.

## Materials and methods

### Participants

Ten healthy young adults without known history of neurological, muscular, or orthopedic problems participated in the study (3 male, age: 21 ± 2 year, height: 1.76 ± 0.1 m, weight: 65 ± 9 kg). The local medical ethics committee (Medisch Ethische Toetsingscommissie Twente) approved the experimental setup and protocol. All participants gave written informed consent prior to the experiment, in accordance with the Declaration of Helsinki.

### Apparatus

**Figure 1:**
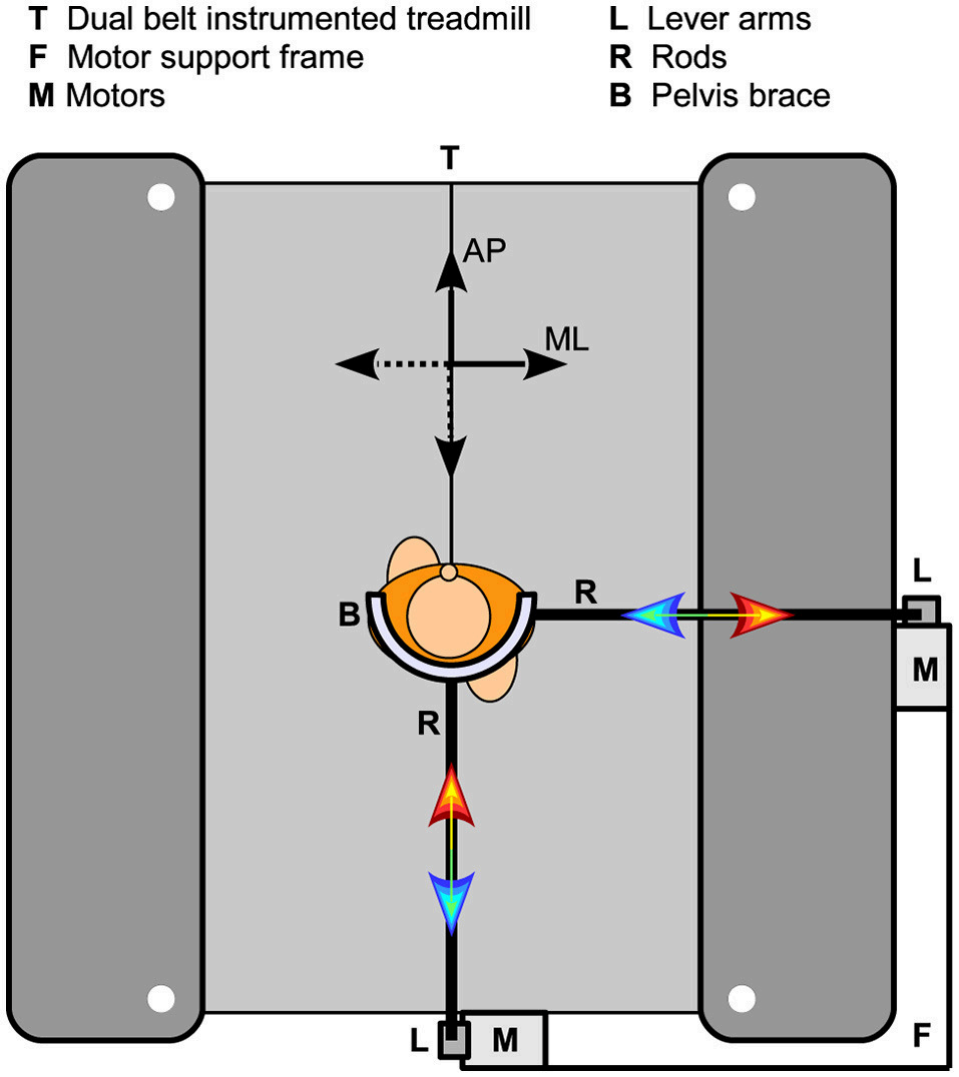
Experimental setup. Two motors were fixated on a support frame, which in turn was attached to a dual-belt instrumented treadmill. Each motor could be connected to the subject through a vertical lever arm, a horizontal rod, and a hip brace. The two motors were never attached to the subject at the same time.

Here only a brief description of the experimental setup is given. A more detailed description is provided elsewhere (Vlutters et al., 2016). A dual-belt instrumented treadmill (custom Y-mill, Motekforce Link, Culemborg, The Netherlands) and two motors (SMH60, Moog, Nieuw-Vennep, The Netherlands) adjacent to the treadmill were used to deliver ML and AP pelvis perturbations during walking in a controlled way, see Figure 1. Attached to each motor was a vertical lever arm, which in turn was connected to a horizontal rod through a ball-joint. The rod was connected to a hip brace (universal hip abduction brace, Distrac Wellcare, Hoegaarden, Belgium), also using a ball joint. The brace was worn by the subject. Control signals for the motors were generated using xPC-target (MathWorks, Natick, MA, USA) at 1,000 Hz.

### Data collection

Subject kinematic data of the feet, lower legs, upper legs, pelvis, upper body, and head were captured using a 9-camera motion capture system (Visualeyez II, Phoenix Technologies, Burnaby, Canada). To this purpose a three-LED cluster was attached to each of those body segments. Additional single LEDs were placed on both lateral malleoli, and both lateral epicondyle of the femur. The torque and angle of each motor in the perturbation device were collected over UDP using an Ethernet card (82558 Ethernet card, Intel, Santa Clara, CA, USA). Ground reaction force data of the treadmill were also collected at 1,000 Hz using an AD card (PCI-6229, National Instruments, Austin, TX, USA). Both cards were part of the xPC-target hardware. The AD card was also used to generate an analog signal to synchronize the motion captures system with the xPC-target hardware.

### Protocol

Before the start of the experiment, several kinematic measurements were performed during which the locations of the bilateral first and fifth metatarsal heads, calcaneus, medial and lateral malleoli, fibula heads, medial and lateral epicondyles of the femur, greater trochanter, anterior and posterior superior iliac spines, xiphoid process, jugular notch, 7th cervical vertebra, occiput, head vertex, and nasal sellion were indicated using an LED-based probe (Cappozzo et al., 1995), relative to the LED clusters on each body segment. Using these measurements and the measured global positions of the LED clusters, the indicated points can be reconstructed in global space throughout all measurements.

During the experiment, subjects walked on the treadmill with their arms crossed over the abdomen, to prevent balancing using the arms. The walking speed was 0.63 m s^−1^ multiplied with the square root of the subject's leg length (Hof, 1996). Subjects walked two blocks of five trials each. The first trial of each block was a 2-min baseline trial in which no perturbations were applied. The first baseline trial was furthermore used to determine the single support duration during unperturbed walking. The remaining four trials were perturbation trials. A perturbation consisted of a sudden square-wave pulse with a duration of 150 ms. Perturbation onset occurred at right toe-off, at the start of the left single support phase (SS_0_), at one third of the left single support phase (SS_1/3_), and at two thirds of the left single support phase (SS_2/3_). The interval between subsequent perturbations was random, between 6 and 12 s. The delivered force magnitudes were equal to 8 and 16 percent of the subject's body weight. Perturbations were directed either inward (negative sign, leftward for right swing) and outward (positive sign, rightward for right swing), or forward (positive sign) and backward (negative sign). In one block only ML perturbations were applied, in the other block only AP perturbations. Block order was randomized across subjects. Within a block, all perturbations were randomized over onset instance, magnitude, and direction. Each condition was repeated 8 times, yielding 196 perturbations in total per subject. When no perturbation force was being delivered, the interaction force between subject and motor was regulated to (near) zero using admittance control, which allowed the subject to move freely. Subjects wore a safety harness at all times to prevent the body from hitting the treadmill in case of a fall.

### Data processing

Data were processed using Matlab (R2016b, MathWorks, Natick, MA, USA). Marker data were filtered with a 4th order zero-phase 20 Hz low-pass Butterworth filter. Landmark positions were subsequently reconstructed using the probe measurements through least squares estimation (Söderkvist and Wedin, 1993). Using a method comparable to that in Zeni et al. (2008), the calcaneus and first metatarsal head landmarks on both feet were used to detect gait phase events of toe-off right (TOR), heel strike right (HSR), toe-off left (TOL), and heel strike left (HSL). All landmark data was used to estimate the location of the COM of each segment, as well as that of the whole body COM (Dumas et al., 2007). The COM position was differentiated to find COM velocities.

The unperturbed walking data from the baseline trials was used to find the average Euclidean distance between the COM of the feet at heel strike. This value was used as a scaling factor (*l*_0_) to make all position and velocity data dimensionless following (Hof, 1996). Next, all position and velocity data were expressed relative to those of the whole body COM. The velocity of the whole-body COM itself was expressed relative to the treadmill belt. All data were sorted on perturbation magnitude, direction, and onset. The COM velocity data was cut into sequences using the gait phase events. Each sequence was resampled to 50 samples to allow averaging across repetitions and subjects. All data at gait events were averaged over repetitions to obtain average data per subject. These were used to obtain subject averages and standard deviations.

To investigate the predictive power of the COM velocity on the COP location, linear least squares fits were made to the distance between the COP and the COM at TOL as a function of the COM velocity at the preceding HSR, in line with our previous analysis (Vlutters et al., 2016). Such fits have previously shown to correspond well to the XCOM concept, with the COP-COM distance proportional to the COM velocity times a factor $\omega_{0}^{-\text{1}}$ = √(*l*/*g*), in which *l* is the subject's leg length, and *g* is the Earth's gravitational acceleration. A dimensionless XCOM proportionality constant ($\omega_{0}^{-\text{1}}$) was calculated for each subject, and averaged over all subjects for comparison with the fits. This constant was compared to the slope of the linear fits to the data. If both are similar, the COP modulates in a comparable way with the COM velocity as the XCOM does, for specific instances in the gait cycle.

Linear mixed models were used to assess the effect of the perturbation (fixed factor, with intercept) and the onset timing (fixed factor, with intercept) on the ML and AP distance between the COM and the COP at TOL, as well as on the duration of the single and double support phases during and after the perturbation. Subject effects were included as a random factor (intercept) to account for correlation effects from repeated measures within the same subject. A significance level of α = 0.05 was used and a Bonferroni correction was applied to correct for multiple comparisons during *post hoc* analysis. The perturbed conditions were only compared to the unperturbed condition and not mutually to reduce the number of comparisons. Finally, the analysis was performed separately for ML and AP perturbations. SPSS statistics 21 (IBM Corporation, Armonk, NY, USA) was used for the statistical analysis.

## Results

Foot placement adjustments in terms of location and time were assessed following both ML and AP perturbations in walking subjects. All data are shown dimensionless. Subject-average scaling factors to make the data dimensionless were *l*_0_ = 0.41 ± 0.03 m for distances, √(*g* ^*^ *l*_0_) = 2.02 ± 0.07 m s^−1^ for velocities, and √(*l*_0_ / *g*) = 0.21 ± 0.01 s for durations, where *l*_0_ is the average Euclidean distance between the COM of both feet at unperturbed heel strike.

For various ML perturbations one subject showed stepping strategies that were not consistent with the other subjects. These special cases are shown separately in **Figures 4**–**6**, and were removed from the statistical analysis. For the 0.16 magnitude outward perturbation applied at SS_2/3_ the responses were not consistent across subjects, nor within several subjects. As a result, the data cannot be pooled subject-wise to represent a specific strategy. Corresponding data were omitted from **Figures 4**, **6** to prevent image cluttering, and were also removed from the statistical analysis. However, all data were included when determining the relations of the COP data with the COM velocity. As we have previously demonstrated in Vlutters et al. (2016), the underlying COP might still modulate with the COM velocity, in line with the XCOM concept, throughout different balance strategies.

### Perturbation effects on COM velocity

**Figure 2:**
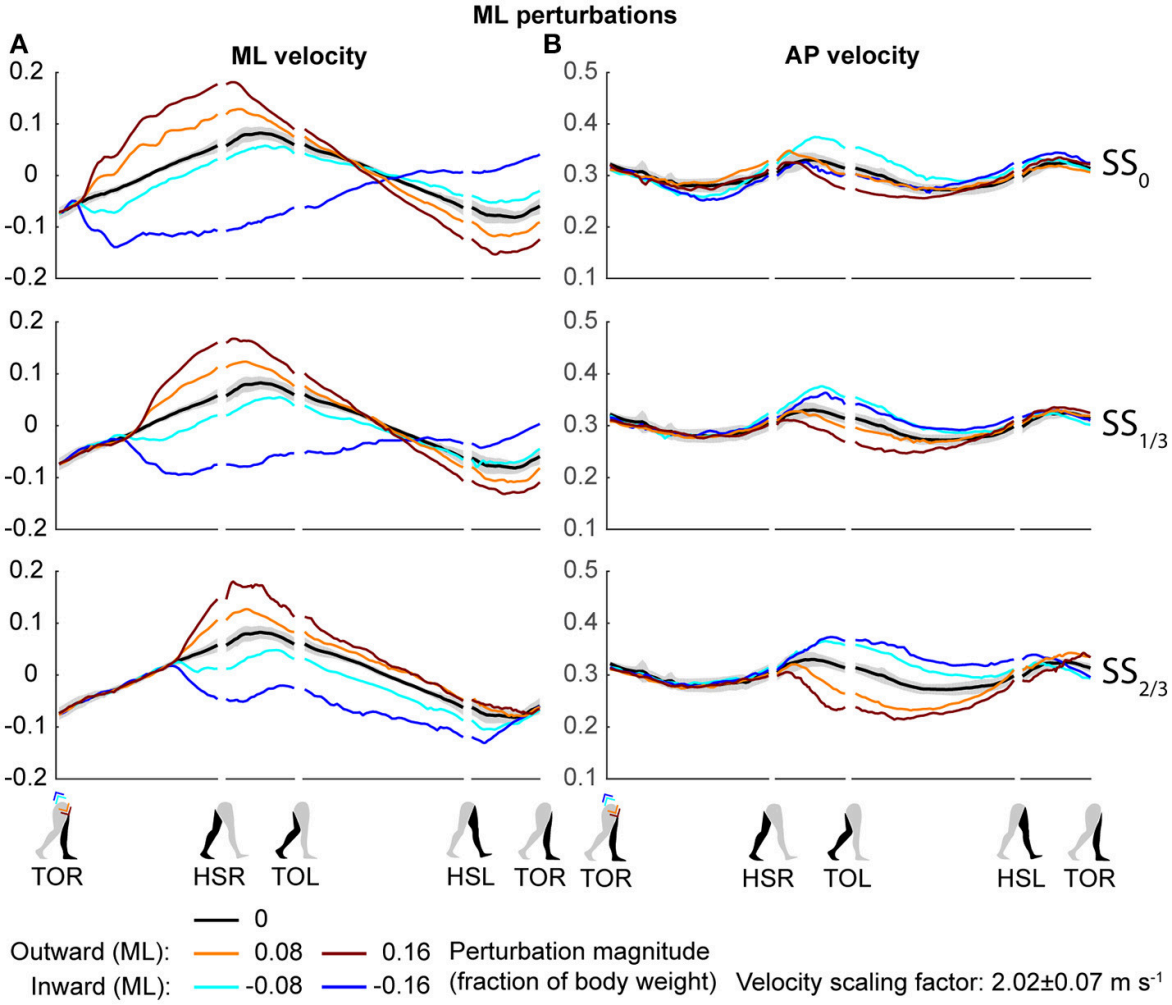
COM velocity profiles following ML perturbation. **(A)** ML COM velocity in response to ML perturbations. **(B)** AP COM velocity in response to ML perturbations. Top, middle, and bottom rows correspond with perturbation onset instances of SS_0_, SS_1/3_, and SS_2/3_, respectively. Data is shown as a function of the gait phase. Colors indicate the various perturbation magnitudes. Shaded areas indicates the subject-standard deviation for the unperturbed condition. It is not shown for perturbed conditions to prevent image cluttering. Data is shown dimensionless.

**Figure 3:**
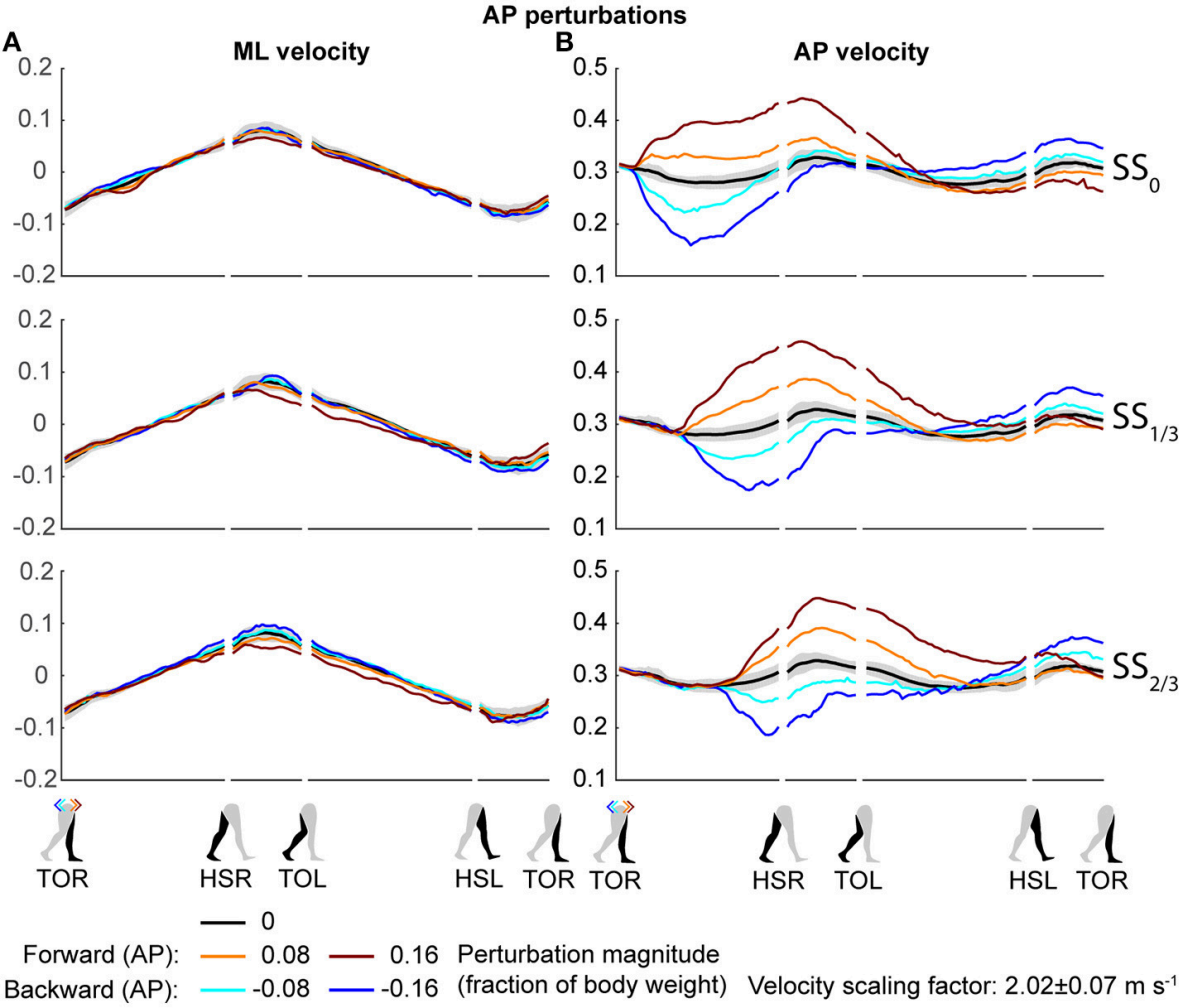
COM velocity profiles following AP perturbation. **(A)** ML COM velocity in response to AP perturbations. **(B)** AP COM velocity in response to AP perturbations. Top, middle, and bottom rows correspond with perturbation onset instances of SS_0_, SS_1/3_, and SS_2/3_, respectively. Data is shown as a function of the gait phase. Colors indicate the various perturbation magnitudes. Shaded areas indicates the subject-standard deviation for the unperturbed condition. It is not shown for perturbed conditions to prevent image cluttering. Data is shown dimensionless.

Both the ML and AP perturbations affected the subject's COM velocity, see Figures 2, 3. The velocity profiles following ML perturbations appear dependent on the onset timing. Deviations from the unperturbed case obviously start later for later perturbation onset, but the way the velocity progresses changes with the onset timing as well. The effects of various AP perturbations on the COM velocity appear less dependent on the onset timing. Though later onset leads to later deviations from the unperturbed case, the velocity profiles between the different onset conditions start to appear similar again at HSL, at the start of the second step.

The disturbances move the subjects in the direction of the perturbation, such that subjects had to return to the center of the treadmill during their recovery. This return to the center can be derived from the velocity data, corresponding to the instances where the perturbed velocity data crossed the unperturbed velocity data, see Figure 2A. The point at which this return occurs following ML perturbations shifts with the perturbation onset, becoming later for perturbations that are applied later. This is less the case for AP perturbations, see Figure 3B. Finally, the COM velocity perpendicular to the perturbation direction remains relatively unaffected by the perturbation itself, but may change through subject actions following HSR. This is mainly the case for ML perturbations, especially for those with SS_2/3_ onset, see Figure 2B. Subjects speed up in the walking direction for inward perturbations, and slow down for outward perturbations.

### Foot placement location following ML perturbations

**Figure 4:**
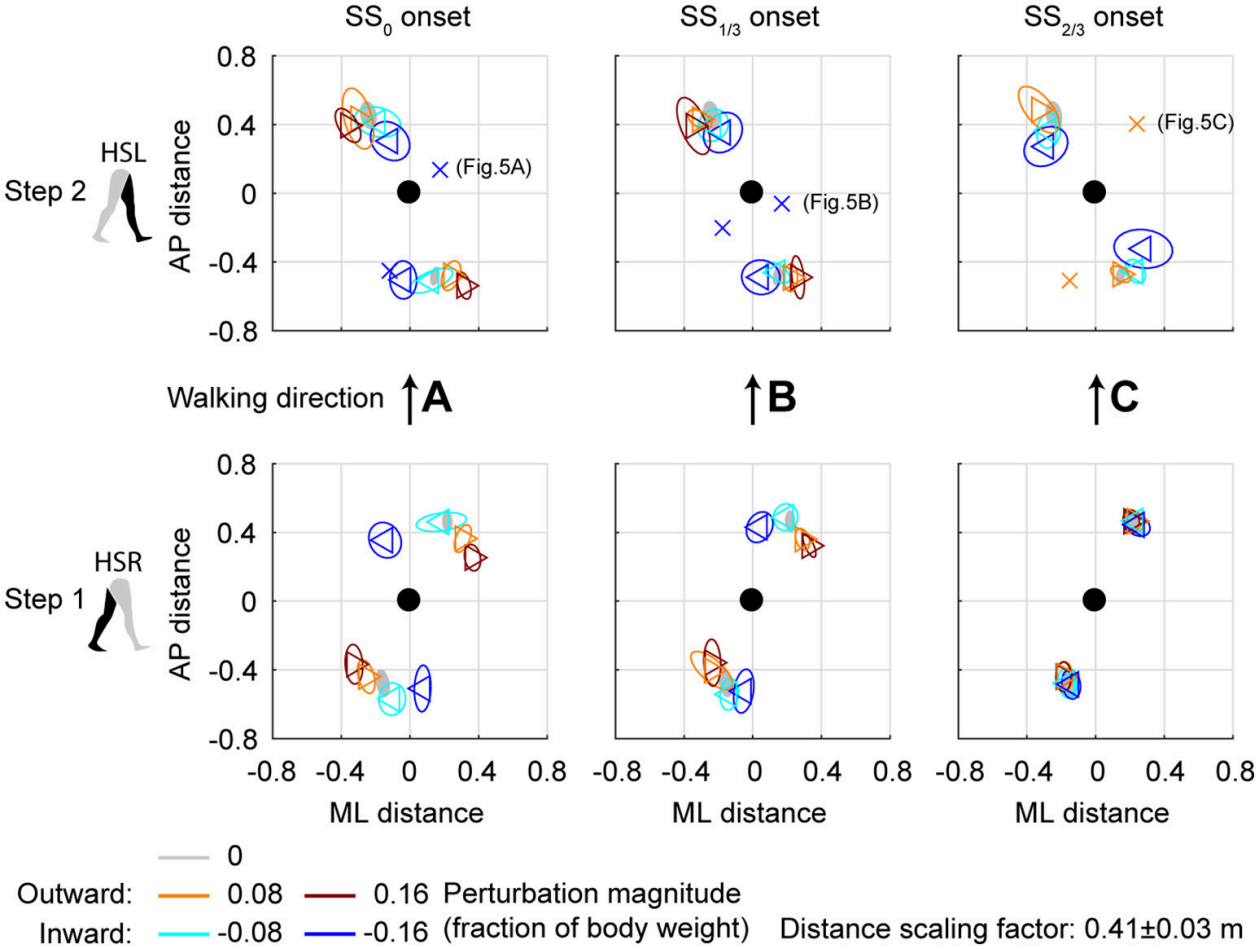
Foot locations following ML perturbations. **(A)** Top-down view of the locations of the COM of the leading and trailing feet relative to the whole body COM at (0,0). Perturbation onset was at SS_0_. Bottom plot corresponds to the first step (HSR), top plot corresponds to the second step (HSL) after the perturbation. For the second step, one subject deviated from the other subjects. The repetition-average data of this subject is given by a cross. **(B)** Same as **(A)**, but for perturbation onset at SS_1/3_. **(C)** Same as **(A)**, but for perturbation onset at SS_2/3_. The subject averages for the 0.16 magnitude SS_2/3_ perturbation are not shown as it is not representative of any specific stepping strategy, but contains a mixture of three different strategies. Triangles show subject-averages and correspond to the perturbation direction. Ellipses represent the subject-standard deviation. Colors indicate the different perturbation magnitudes. Data is shown dimensionless.

Subjects modulated their foot placement in terms of location and/or timing following the perturbations. Especially ML perturbations with SS_0_ onset lead to adjustments in foot placement location in the first recovery step, see Figure 4. This might be expected given that these perturbations are perpendicular to the walking direction, while there is sufficient time to adjust the foot. Note that the locations of the feet in Figure 4 are represented *relative* to the COM. The location of the leading foot relative to the COM results from the step. The location of the (mostly stationary) trailing foot can change relative to the COM because the COM itself moves as a result of the perturbation. In the first step, at HSR, subjects generally placed their foot in the direction of the perturbation at an increased ML distance from the COM with increasing ML perturbation magnitude. The ML distance between the COM and the leading foot was significantly affected by the ML perturbations [*F*_(4, 126)_ = 114.410, *p* < 0.001], the onset timing [*F*_(2, 126)_ = 7.605, *p* = 0.001], and their interaction [*F*_(8, 126)_ = 126.000, *p* < 0.001]. However, the *post-hoc* analysis revealed that the leading foot was not placed significantly different from the unperturbed case for any ML perturbation with SS_2/3_ onset (*p* = 1.000). The main effect of perturbation on the ML distance between leading foot and COM is therefore mainly caused by the SS_0_ and SS_1/3_ onset perturbations. Other exceptions were the −0.08 inward perturbations with SS_0_ and SS_1/3_ onset, which also did not lead to significant changes in this distance (*p* ≥ 0.589).

For the second step, at HSL, modulation of the foot location occurred, but the changes in ML distance between the leading foot and the COM tend to diminish for perturbations with a later onset. A possible explanation is that part of the recovery occurred during the double support phase between the first and second step, and the single support phase prior to foot contact of the second step. This way, there is less need for adjustments in the location of the foot. Note that the 0.16 magnitude outward perturbations with SS_2/3_ onset are disregarded here, for which lateral foot adjustments did occur. For perturbations with SS_0_ and SS_1/3_ onset, the ML distance to the leading foot in the second step deviates from the unperturbed condition for various reasons, such as uncrossing the legs following a cross-step, or to return to the center of the treadmill. The ML distance between the COM and the leading foot in the second step was significantly affected by the perturbations [F_(4, 114.101)_ = 23.251, *p* < 0.001], the onset timing [*F*_(2, 114.068)_ = 9.262, *p* < 0.001], and their interaction [*F*_(7, 114.058)_ = 2.825, *p* = 0.009]. Visual inspection of Figure 4 suggests that the modulation pattern as seen in the first step in response to ML perturbations with SS_0_ onset do not clearly re-appear in the second step for ML perturbations with SS_2/3_ onset, which suggests recovery occurs before foot contact of the second step, even if there was no foot adjustment in the first.

### Alternative foot locations following ML perturbations

**Figure 5:**
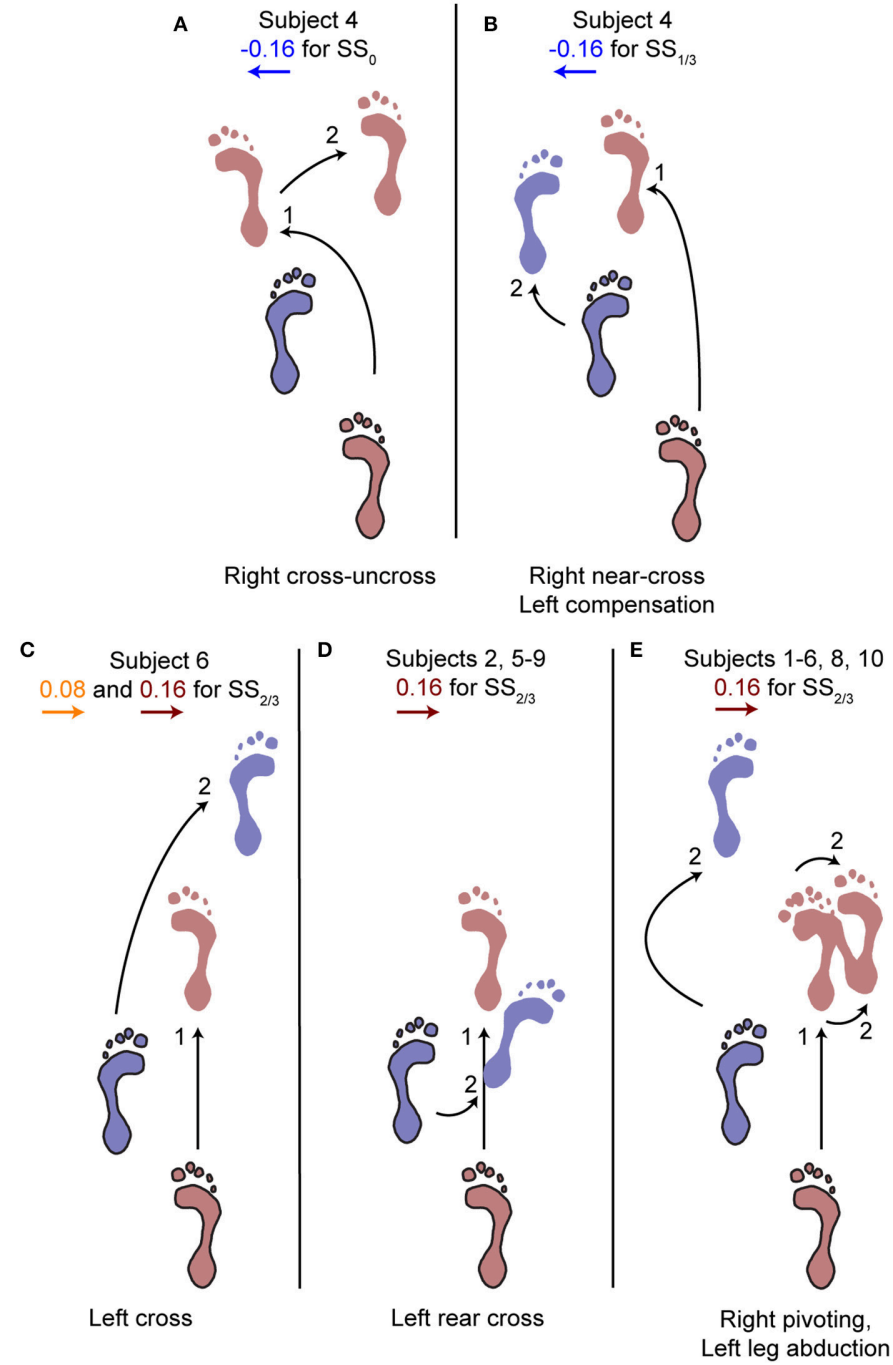
Alternative stepping strategies. Alternative strategies used by one or multiple subjects for specific perturbations. The number (1, 2) corresponds to the step number in Figure 4. **(A)** Cross-uncross, making a double step with the right leg. **(B)** Near-cross step with the right leg, followed by a short left step to prevent the body from falling leftward. **(C)** Left cross-step. **(D)** Rear cross-step. **(E)** Foot pivoting, by first rotating about the toes, then shifting the COP back toward the heel and rotating about the heel. Accompanied by left leg abduction during the swing to prevent toppling over the right stance leg. Several subjects showed both **(D,E)** throughout the repetitions of the 0.16 perturbations at SS_2/3_.

**Figure 6:**
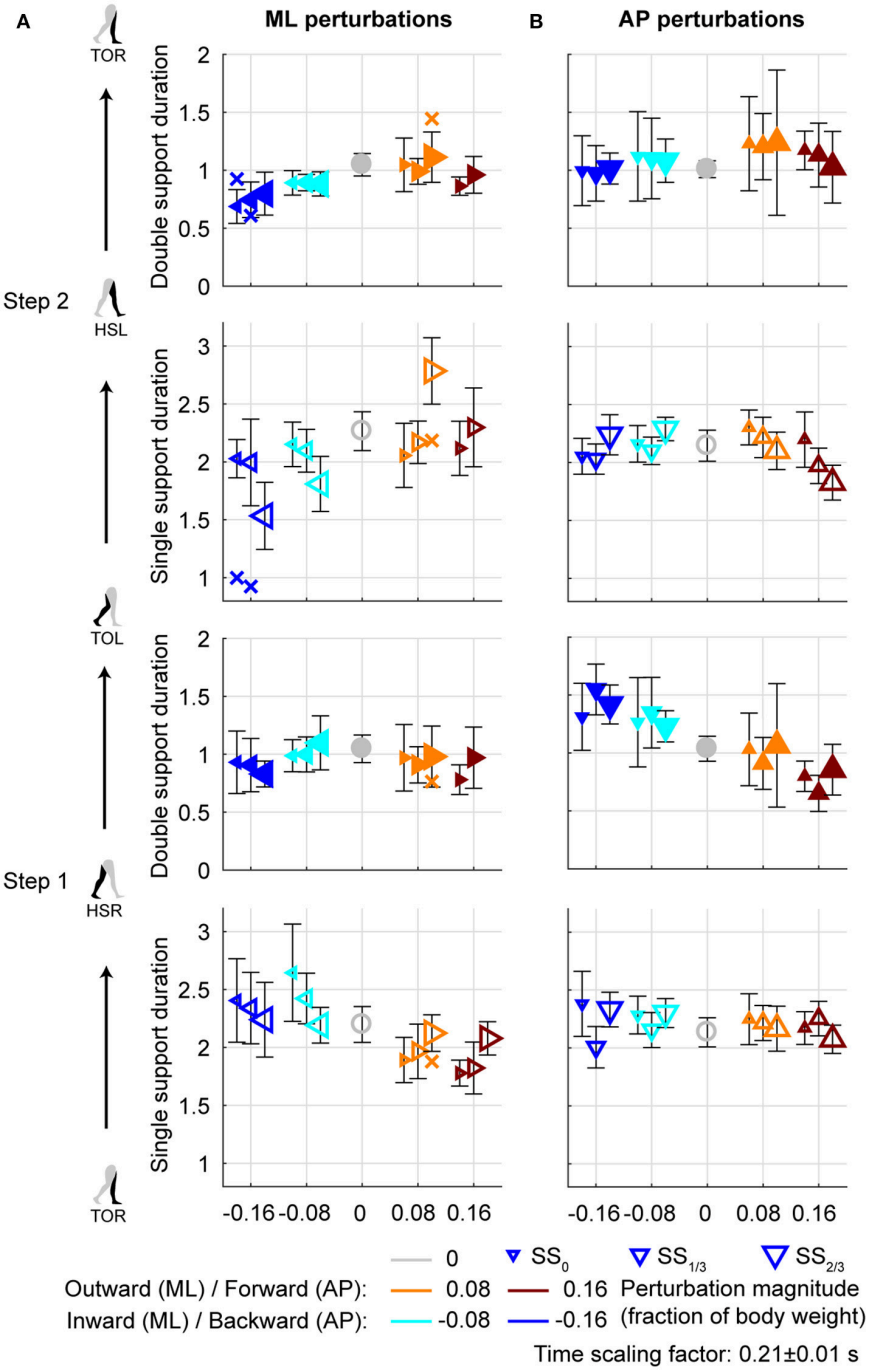
Gait phase durations. **(A)** Gait phase durations following ML perturbations. **(B)** Gait phase durations following AP perturbations. The individual subjects with an alternative strategy for step 2 are shown separately with a cross. Triangles show subject-averages and indicate the perturbation direction. Larger marker size corresponds with later perturbation onset. Error bars represent the subject-standard deviation. Open and filled markers correspond to the single and double support phases, respectively. Colors indicate the different perturbation magnitudes. Data is shown dimensionless.

The aforementioned statistical results do not include the alternative strategies performed by some subjects in the second step, deviating from the rest of the population, see Figure 5. For perturbations with SS_0_ and SS_1/3_ onset, one subject showed alternative stepping responses for a specific perturbation. Subject 4 consistently performed a double right step following the −0.16 inward perturbations with SS_0_ onset, first crossing the legs in the first step like all other subjects, but then uncrossing the legs with a second right step (Figure 5A). Furthermore, subject 4 made a consistent short compensatory step with the left leg during the second step after the −0.16 inward perturbations with SS_1/3_ onset, to further counteract the induced inward COM velocity (Figure 5B). This short step was also of shorter duration than that of the other subjects (Figure 6).

For perturbations with SS_2/3_ onset more varying responses occurred, especially for the 0.16 outward perturbations. Subjects 1, 3, 4, and 10 performed left leg abduction, opposite of the perturbation direction, as well as foot pivoting. Moving the heel laterally by pivoting on the forefoot, and subsequently moving the forefoot laterally by pivoting on the heel allows changes in the base of support using only a single foot, without actual stepping (Figure 5E). Subjects 7 and 9 performed a rear cross-step (Figure 5D), stepping behind the leading leg without the body fully toppling over the leading foot in the sagittal plane. Subjects 2, 5, and 8 performed both of these strategies, and subject 6 even performed three different strategies, including a cross-step using the left leg (Figure 5C). Furthermore, subject 6 was the only subject to perform such a cross-step for the 0.08 outward perturbations with SS_2/3_ onset. Other subjects dealt with this perturbation through a relatively long lasting right single support phase during which the left leg was abducted, sometimes combined with foot pivoting as in Figure 5E. The leg abduction is not directly clear from the foot placement locations, but is in line with the long lasting single support duration of the second step following 0.08 outward perturbations with SS_2/3_ onset in Figure 6.

### Foot placement timing following ML perturbations

Aside from adjustments in foot placement location, the gait phase durations following the perturbations were affected as well, see Figure 6A. For the first step, inward perturbations tend to increase the single support duration, whereas outward perturbations tend to decrease it. These effects diminish with later perturbation onset. In contrast, for the second step inward perturbations tend to decrease the single support duration, whereas outward perturbations tend to increase it. These effects become stronger with later perturbation onset. Specifically, major deviations occur for the −0.16 inward and the 0.08 outward perturbations with SS_2/3_ onset. For the −0.16 inward perturbation a fast step with the left leg is used to correct in the second step. For the 0.08 outward perturbation the duration increases because of the earlier mentioned leg abduction strategy that occurs during this single support phase.

All gait phase durations were affected by the ML perturbations [*F*_(4, 144.053)_ ≥ 5.248, *p* ≤ 0.001], but there was no main effect of the onset timing on any of the gait phases [*F*_(2, 114.032)_ ≤ 1.585, *p* ≥ 0.209]. This is likely because the effects of inward and outward perturbations tend to cancel out in the average duration over all perturbations. A significant interaction effect was found only for the single support phases of the first and second step [*F*_(7, 114.025)_ ≥ 9.069, *p* ≤ 0.001], but not for any of the double support phases [*F*_(7, 114.127)_ ≤ 1.269, *p* ≥ 0.166]. For the single support phase of the first step, deviations in duration from the unperturbed case diminish with increasing perturbation onset delay. For the ML perturbations with SS_2/3_ onset there were no significant differences from the unperturbed case (*p* > 421). This is consistent with the findings for the foot locations in the first step following SS_2/3_ onset perturbations (Figure 4C). It is likely that subjects cannot make major adjustments to their foot placement if the remaining time to the intended (unperturbed) foot contact is short.

### Foot placement location following AP perturbations

**Figure 7:**
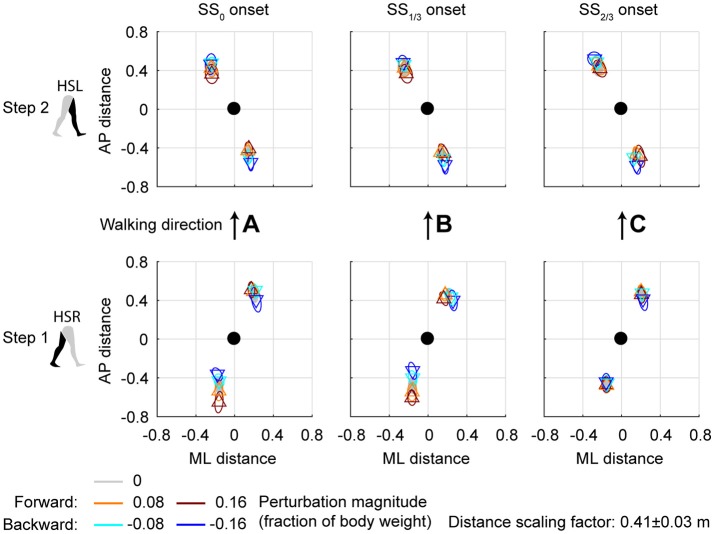
Foot locations following AP perturbations. **(A)** Top-down view of the locations of the COM of the leading and trailing foot relative to the whole body COM at (0,0). Perturbation onset was at SS_0_. Bottom plot corresponds to the first step (HSR), top plot corresponds to the second step (HSL) after the perturbation. **(B)** Same as **(A)**, but for perturbation onset at SS_1/3_. **(C)** Same as **(A)**, but for perturbation onset at SS_2/3_. Triangles show subject-averages and correspond to the perturbation direction. Ellipses represent the subject-standard deviation. Colors indicate the different perturbation magnitudes. Data is shown dimensionless.

Changes in the AP distance between the COM and the leading foot during both the first and second step after the AP perturbations are generally small, see Figure 7. For forward perturbations there is a tendency for the first step to be longer and the second to be shorter, while the opposite is the case for backward perturbations. The leading-foot AP distance from the COM was significantly affected by the AP perturbations [***F***_(4, 126)_ ≥ 9.252, *p* < 0.001] and the onset timing [*F*_(2, 126)_ ≥ 7.432, *p* = 0.001], but not by their interaction [*F*_(8, 126)_ ≤ 1.600, *p* ≥ 0.131], for both at HSR (step 1) and HSL (step 2). Unlike the results in Vlutters et al. (2016), here this AP distance was significantly affected by the perturbations. However the differences are generally small, with a typical mean difference of 1 cm between the various onset conditions, as well as between the various AP perturbations and the unperturbed condition. The foot locations in step 2 appear similar regardless of the onset timing. This suggest that the AP perturbations are mostly rejected during the double support phase following the disturbance. No alternative strategies were observed in response to AP perturbations.

### Foot placement timing following AP perturbations

For AP perturbations of any onset timing, the most prominent changes in gait phase duration seem to occur in the double support phases rather than in the single support phases, see Figure 6B. All gait phase durations were affected by the AP perturbations [*F*_(4, 126)_ ≥ 5.168, *p* ≤ 0.001]. For the first double support phase, backward perturbation leads to increases in duration, and forward perturbations to decreases. The second double support phase has the tendency to show opposite effects. This is possibly related to the distance between the COM and the trailing foot at heel strike. If this distance is larger, the trailing foot will have to leave the floor earlier during the subsequent double support phase, making it of shorter duration. When considering the perturbation onset timing, only the single support durations were affected [*F*_(2, 126)_ ≥ 4.827, *p* ≤ 0.010] but not the double support durations [*F*_(2, 126)_ ≤ 0.310, *p* ≥ 0.712]. Most gait phase durations were also affected by the interaction effect [*F*_(8, 126)_ ≥ 2.112, *p* ≤ 0.039], with exception of the double support phase of step 2 (HSL-TOR). This indicates that the perturbation responses in this double support phase are independent of the onset timing of the perturbation. The effects of the perturbation onset must therefore have been negated in an earlier gait phase.

### Relations with the COM velocity

**Figure 8:**
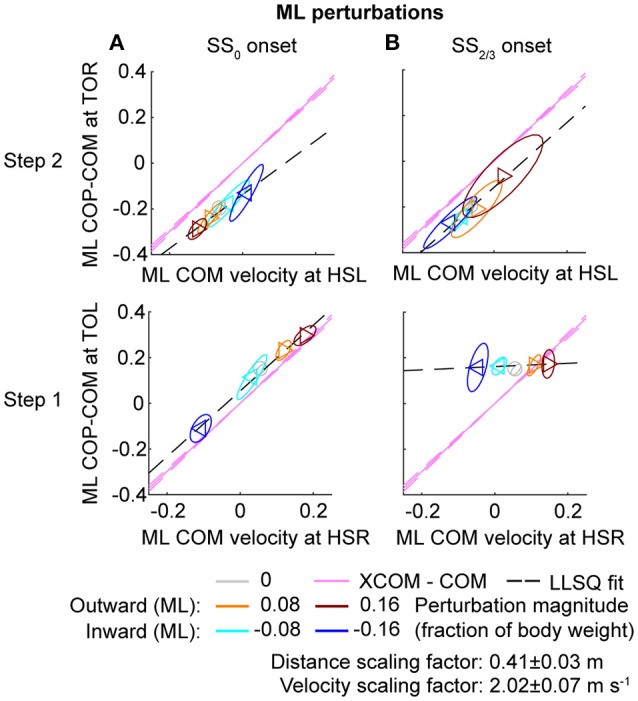
Relation COM velocity and COP location in comparison with the XCOM, following ML perturbations. **(A)** The ML COP location relative to the COM, at TOL (step 1) and TOR (step 2) as a function of the ML COM velocity at the preceding heel strike. Perturbation onset was at SS_0_. **(B)** Same as **(A)**, but for perturbation onset at SS_2/3_. The pink line corresponds to the XCOM position relative to the COM, as a function of the COM velocity. It has slope $\omega_{0}^{-1}$ and zero intercept. The pink dashed lines indicate the between-subject standard deviation of the XCOM, based on the differences in leg length between subjects. The black dashed line is a linear least squares fit to the data. Triangles show subject-averages and indicate the perturbation direction. Ellipses represent the subject-standard deviation. Colors indicate the different perturbation magnitudes. Data is shown dimensionless.

**Figure 9:**
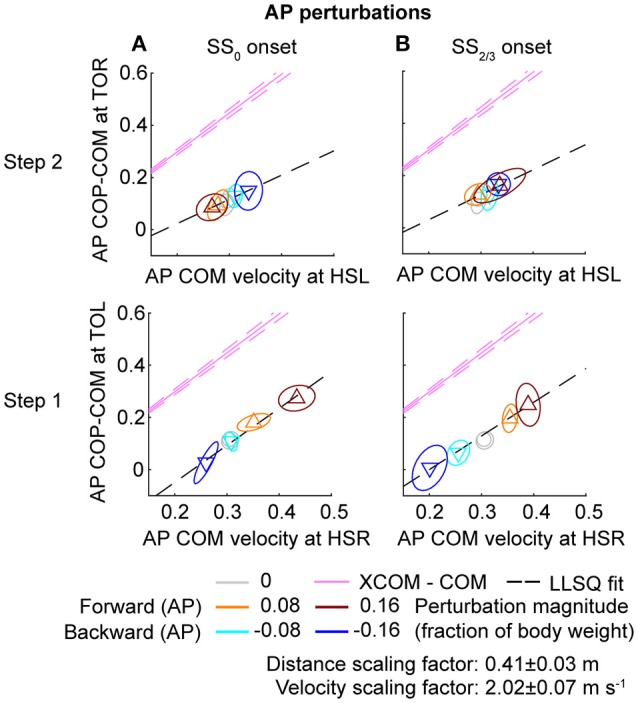
Relation COM velocity and COP location in comparison with the XCOM, following AP perturbations. **(A)** The AP COP location relative to the COM, at TOL (step 1) and TOR (step 2) as a function of the AP COM velocity at the preceding heel strike. Perturbation onset was at SS_0_. **(B)** Same as **(A)**, but for perturbation onset at SS_2/3_. The pink line corresponds to the XCOM position relative to the COM, as a function of the COM velocity. It has slope $\omega_{0}^{-1}$ and zero intercept. The pink dashed lines indicate the between-subject standard deviation of the XCOM, based on the differences in leg length between subjects. The black dashed line is a linear least squares fit to the data. Triangles show subject-averages and indicate the perturbation direction. Ellipses represent the subject-standard deviation. Colors indicate the different perturbation magnitudes. Data is shown dimensionless.

In line with previous studies (Hof et al., 2010; Vlutters et al., 2016) we investigated the predictive value of the COM velocity for the location of the COP after foot contact. Note that the relations presented here span two instances of the gait cycle: heel strike for the velocity, and the subsequent toe-off for the COP. We have previously found this to provide the best correspondence with the XCOM concept (Vlutters et al., 2016), which is also dependent on the COM velocity. The slopes, intercepts, and coefficients of determination of the fits to the data are presented in Table 1. If the data modulates with the same slope ($\omega_{0}^{-\text{1}}$) as that of the pink XCOM line in Figures 8, 9, then the COP shifts to a constant offset from the XCOM on average over all perturbation magnitudes. If this is the case, the XCOM plus an offset might be used as predictor for the COP location. The subject-average dimensionless XCOM proportionality constant $\omega_{0}^{-\text{1}}$ was 1.49 ± 0.05, for comparison with the slopes in Table 1.

**Table 1:** Slope, intercept, and coefficient of determination of the linear least squares (LLSQ) fit made to the subject-average data at specific instances of the gait cycle after the perturbation.

**ML PERTURBATIONS**			
	**ML COM velocity, at HSR (step 1)**		
**ML distance COP-COM at TOL (step 1)**	**Slope**	**Intercept**	***R*^2^**
SS_0_ onset	1.425	0.055	0.994
SS_1/3_ onset	1.284	0.079	0.986
SS_2/3_ onset	0.090	0.159	0.974
	**ML COM velocity, at HSL (step 2)**		
**ML distance COP-COM at TOR (step 2)**	**Slope**	**Intercept**	***R***^2^
SS_0_ onset	1.081	−0.146	0.962
SS_1/3_ onset	0.853	−0.165	0.896
SS_2/3_ onset	1.444	−0.110	0.931
**AP PERTURBATIONS**			
	**AP COM velocity, at HSR (step 1)**		
**AP distance COP-COM at TOL (step 1)**	**Slope**	**Intercept**	***R***^2^
SS_0_ onset	1.348	−0.314	0.959
SS_1/3_ onset	1.020	−0.212	0.966
SS_2/3_ onset	1.268	−0.255	0.998
	**AP COM velocity, at HSL (step 2)**		
**AP distance COP-COM at TOR (step 2)**	**Slope**	**Intercept**	***R***^2^
SS_0_ onset	1.058	−0.204	0.979
SS_1/3_ onset	1.000	−0.192	0.885
SS_2/3_ onset	0.960	−0.162	0.632

*Distance COP-COM is the independent variable, COM velocity the dependent variable. Underlined values correspond with fits of which the root mean square error is less than 5% of the range of the dependent variable*.

For ML perturbations, both COP and XCOM modulate in a similar way with the COM velocity within the first recovery step if the perturbation is given early, at SS_0_, see Figure 8 and Table 1. Perturbations with a later onset diminish the similarities, in line with the reduced foot location modulation (Figure 4). The limited base of support adjustment prevents such COP modulation. In contrast, for the second step, similarities are highest if the perturbation is given late, at SS_2/3_. If the perturbation is given early instead (SS_0_), most of the balance recovery can occur in the first step, such that the second step can be used to return to the center of the treadmill. This might diminish the similarities with the XCOM. Furthermore, a major contributor to the fit to the data in the second step is the mixture of strategies for the 0.16 outward perturbations at SS_2/3_, see Figure 5B. Even though the standard deviation is large due to the various strategies, its variation is aligned with the XCOM line. Note that for the second step the data is located on the other side of the XCOM line, as the step is made with the other leg. For AP perturbations, both COP and XCOM modulate in a comparable way with the COM velocity within the first step for perturbations with SS_0_ or SS_2/3_ onset, see Figure 9. The similarity is less for perturbations with SS_1/3_ onset (see Table 1). For the second step, too, the modulation in COP and XCOM is less similar compared to step 1, irrespective of the perturbation onset.

## Discussion

The study aim was to investigate how healthy humans deal with balance perturbations if foot placement adjustments are increasingly constrained by time. Walking subjects received both AP and ML pelvis perturbations at various onset instances throughout the single support phase. For AP perturbations the first step is relatively insensitive to the onset timing. For ML perturbations, adjustments in foot placement location and time in the first recovery step clearly diminished with increasing perturbation onset delay. Most adjustments in foot placement were consistent across subjects, with several exceptions in the second step. Mainly the largest magnitude (0.16) outward perturbations with an onset at two-thirds of the left single support phase (SS_2/3_ onset) resulted in inconsistent and varying responses across and within subjects during this second step.

### Balance responses are gait phase dependent

Gait-phase-dependent responses arise because foot placement modulation takes time. Hof and colleagues (Hof et al., 2010) reported at least 0.28 s to be required for a “correct” lateral positioning of the foot to occur, though it is unclear how “correct” was defined. Here, the foot placement location relative to the COM did not significantly alter if ML perturbations were given at SS_2/3_, but did alter if the onset was at SS_1/3_ or earlier. As a result, more than 0.15 s (0.73 dimensionless time units) are required for significant adjustments to be made, but less than 0.3 s.

Adjustments might also occur mechanically without active involvement of the subject. For −0.16 backward perturbations at SS_2/3_ onset, the single support duration was significantly longer than that for the unperturbed condition. For these perturbations, the single support duration during the first step might increase because the body is pulled backward, which could postpone foot contact resulting from a forward fall due to gravity.

Because of the gait-phase dependency, balance responses should be carefully evaluated with respect to the gait and perturbation characteristics at hand. For example, the 0.16 outward perturbation might be additionally challenging if it is applied shortly before the weight transfers to the leading foot, near the end of the swing phase or start of the double support phase. As loading of the leading foot takes more time in slow walking compared to fast walking (Hebenstreit et al., 2015), it is possible that slow walking is more prone to this specific perturbation. This would contrast with previous indications that slow walking is generally more stable than fast walking, based on the analysis of kinematic variability in unperturbed gait (Dingwell and Marin, 2006; England and Granata, 2007). Because the onset instance within the gait phase can affect how threatening a specific perturbation is, and because the occurrence of gait phases alters with walking speed, one walking speed cannot be declared strictly more stable than another.

### Lack of foot placement adjustments in the first recovery step elicits other strategies

It is not always straightforward to group the balance responses into specific strategies. Though balance control is sometimes divided in ankle, hip, and foot placement strategies (Horak and Nashner, 1986; Maki and McIlroy, 1997), it is certainly not limited to these. For example, when adjustments to the foot location are not possible, foot pivoting allows one-legged base of support corrections without making an actual step. Furthermore, changes in foot placement location and/or time are not necessarily the result of a foot placement strategy. Changes can also be the result of another strategy that occurred before foot contact. The increased single support duration in the second step following the 0.08 magnitude outward perturbations with SS_2/3_ onset is an example of this. Subjects abducted their left swing leg possibly as an inertial strategy, or to provide a counter-weight and shift the whole-body COM in the direction of the perturbation. Because subjects spend time abducting their leg, the change in step time is not strictly because it was required for a specific foot placement adjustment. Hence, possible interaction with other strategies must not be disregarded.

It remains unclear why some subjects prefer one recovery strategy over another, and why alternative strategies mainly occur at higher magnitudes. In Hof et al. (2010) no different strategy uses between or within subjects were reported for a given instant of perturbation within the gait cycle. This is possibly due to the lower perturbation magnitudes. In general, subjects might have less experience dealing with large magnitude disturbances compared to lower ones. Large magnitude perturbations occur less in daily life, such that differences are more likely to arise when the perturbation magnitude is high. The fact that different strategies occurred not only across subjects but also within subjects suggests it is not simply a matter of subject preference. Still, biomechanical constraints could provide some insight on the underlying causes. Whether a cross-step occurs behind or in front of the other leg following outward perturbations at SS_2/3_ likely relates to the AP velocity relative to the ML velocity. That is, if the ML disturbance is so large that it would take too long to traverse over the stance foot in the AP direction to make a corrective cross-step, a backward cross-step might be a preferred option instead. Future experiments might point out if this is indeed the case, for example by combining ML and AP perturbations.

### Various responses contribute to the same relation

The relations between the COM velocity at heel strike and the COP distance from the COM at the subsequent toe-off, previously described in Vlutters et al. (2016), remained intact throughout various conditions. If the perturbations are given early, at SS_0_, the results are comparable with those in our previous study. However, the relation tend to diminish in the second step, possibly because the return to the center of the treadmill begins to play a role. If the perturbation is given late, at SS_2/3_, the relation did not occur in the first step for ML perturbations due to the lack of foot placement adjustments, but re-appeared in the second step, even throughout the varying strategies for the 0.16 outward perturbation. Only for the SS_1/3_ perturbations the relation appears less in both the first and the second step. For these perturbations there might have been insufficient time to expand the base of support to realize the COP—COM velocity relation in the first step, and it might no longer be required for the second step, because recovery actions occur during single and double support phases before the foot contact as well.

### Effects of treadmill walking

Treadmill walking imposes various constraints on the subject that are not present during overground walking. The treadmill requires the subject to continue walking, the treadmill width is limited (~1 m), and there is little optic flow. A comparison of joint kinematics and ground reaction forces between treadmill and overground walking conditions suggests that differences between the two conditions are within the normal variability of gait at a given speed (Riley et al., 2007). Furthermore, in a study by Zadravec et al. (2017), two similar perturbation devices were used to compare human stepping in response to pelvis perturbations during both treadmill and overground walking conditions. They concluded that the responses in both conditions were similar, such that a treadmill condition is generally preferred given the possibility to continuously measure ground reaction forces. We therefore expect the initial stepping responses to generalize to overground walking. However, the treadmill does impose an implicit “center of the road” on the subject, making them eventually return to the center of the treadmill. We expect this to have little effect on the first recovery step, but subsequent steps might generalize less well due to these effects. In addition, the result might not generalize to different walking velocities. Though responses to perturbations with SS_0_ onset were mostly similar for walking speeds of 0.63 and 1.25 m s^−1^ (Vlutters et al., 2016), slower speeds might show reduced effects of the onset timing. Due to lower limb excursion at lower speeds, the body configuration at perturbation onset would be more similar across the different onsets.

## Conclusions

First, we questioned whether foot placement adjustments diminish when there is little time to use such adjustments as a recovery strategy? Foot placement modulation takes time and therefore diminishes in the first recovery step if little adjustment time is available after a perturbation. Foot placement adjustments do occur for the second step, but the degree of modulation is dependent on the perturbation magnitude, direction, onset timing, and preceding actions. If foot placement modulation is not an option in the first step, actions during subsequent gait phases are addressed as an alternative. This can lead to peculiar balance strategies such as foot pivoting. These strategies can be inconsistent both across and within subjects. Though it remains unclear what causes the use of the various strategies, subject preferences are unlikely given that differences also occur within subjects. Second, we questioned whether the COP will continue to modulate with the COM velocity, in line with the XCOM? Despite the varying strategies, previously observed relations between the COM velocity and the COP location relative to the COM persist. This relation is in line with the XCOM concept (capture point), supporting its use in balance controllers for humanoid robotics. The relation might disappear in the first step and re-appear to the second step if the perturbation is given late in the preceding single support phase. These results suggest that foot placement, like any other balance strategy, is a way of achieving some underlying objective, possibly reflected in the COP location. Further probing human balance through perturbations might help reveal these objectives.

## Data availability

The data supporting the conclusions of this manuscript will be made available by the authors upon request to any qualified researcher.

## Author contributions

MV: contributed to the design of the experimental protocol, to the collection, processing, analysis, and interpretation of the experimental data, and to drafting and revising the manuscript; EV and HvdK: contributed to the design of the experimental protocol, analysis, and interpretation of the experimental data, and to revising the manuscript.

### Conflict of interest statement

The authors declare that the research was conducted in the absence of any commercial or financial relationships that could be construed as a potential conflict of interest.
